# Molecular histomorphometry and the emerging role of transcriptomics in celiac disease diagnosis and therapeutic trials

**DOI:** 10.1080/07853890.2026.2735791

**Published:** 2026-09-25

**Authors:** Keijo Viiri, Chen Chongtham, Markku Mäki

**Affiliations:** ^a^ Faculty of Biochemistry and Molecular Medicine, University of Oulu, Oulu, Finland; ^b^ Celiac Disease Research Center, Faculty of Medicine and Health Technology, Tampere University, and Tampere University Hospital, Tampere, Finland

**Keywords:** Celiac disease, transcriptomics, histomorphometry, molecular diagnostics, biomarkers, clinical trials

## Abstract

**Background:**

Celiac disease (CeD) is an autoimmune enteropathy in which diagnosis and disease monitoring rely on serology and small-intestinal histology. However, conventional histological assessment is semi-quantitative, subject to interobserver variability, and relatively insensitive to subtle mucosal changes, particularly in treated patients or during early disease. Recent advances in transcriptomic technologies have enabled detailed characterization of intestinal mucosal gene expression, revealing disease-specific molecular signatures. This review aims to synthesize current evidence on molecular histomorphometry and to evaluate the emerging role of transcriptomic profiling in improving diagnosis, disease monitoring, and therapeutic trial endpoints in CeD. In addition, we propose practical guidelines and a methodological pipeline for implementing transcriptomic analyses in this context.

**Discussion:**

Transcriptomic analyses consistently identify characteristic epithelial and immune activation signatures in active CeD, including interferon-driven pathways, epithelial stress responses, and loss of mature enterocyte differentiation. Importantly, persistent molecular abnormalities are often detectable in patients adhering to a gluten-free diet despite histological normalization, highlighting limitations of conventional endpoints. Molecular histomorphometry integrates gene expression data with morphologic features to provide continuous, quantitative measures of mucosal injury. Regression-based molecular indices demonstrate improved sensitivity, reproducibility, and dynamic range compared with traditional histology. In clinical trials, these approaches have potential to enable detection of treatment effects with smaller sample sizes and shorter study durations, and they have shown particular promise as endpoints in gluten challenge models and studies of therapies such as transglutaminase 2 inhibitors. We further outline a practical analytical pipeline, including study design considerations, data processing, model construction, and reporting standards

**Conclusions:**

Molecular histomorphometry and transcriptomic profiling represent a significant advancement in the assessment of CeD, offering quantitative, qualitative and highly sensitive measures of mucosal pathology. Coupled with clear methodological guidance, these approaches have the potential to complement or, in selected contexts, surpass traditional histological evaluation in both clinical practice and drug development. Continued validation, harmonization, and standardization will be essential for broader implementation and regulatory acceptance.

## KEY MESSAGES

- Molecular histomorphometry provides an objective, quantitative, qualitative and reproducible assessment of intestinal mucosal injury, bypassing major shortcomings of conventional histomorphometry such as interobserver variation, subjective scoring, and sampling-related inconsistencies.
- Transcriptomic signatures detect persistent mucosal abnormalities even in histologically healed patients.
- Molecular endpoints can substantially improve sensitivity and efficiency in celiac disease therapeutic trials.

## Background

### Overview of celiac disease

Celiac disease (CeD) is a systemic autoimmune condition triggered by dietary gluten in genetically susceptible individuals carrying the human leukocyte antigen (HLA) molecules DQ2 or DQ8. The disease is characterized by small-intestinal, both a morphological change resulting in villous atrophy and crypt hyperplasia and inflammation measured as intraepithelial lymphocytosis (IELs). CeD affects approximately 1–1.5% of the global population, with considerable geographical variation, and prevalence in Finland exceeds 2%. Clinical presentation has evolved from classical malabsorption towards a broader spectrum of intestinal symptoms and extraintestinal manifestations often identified through serologic case-finding by screening [1–9].

CeD pathogenesis is driven by dietary gluten and transglutaminase 2 (TG2), which deamidates gluten peptides and facilitates their presentation to CD4^+^ T cells *via* HLA-DQ2 or HLA-DQ8 molecules. The resulting immune response promotes intestinal inflammation and progressive mucosal remodelling, characteristic for CeD [3,10].

A lifelong gluten-free diet (GFD) remains the only established treatment. However, inadvertent gluten exposure is common, and complete mucosal recovery is not universal, with quantitative histological studies demonstrating persistent injury in many patients considered clinically well controlled on a gluten-free diet [11–13].

Elderly people may experience a more severe nonresponsive CeD, refractory CeD (RCD) I or II with a manifest small-intestinal enteropathy not reacting to a GFD. These diseases are very rare in the general population and in diagnosed CeD patients. RCD II has a substantial risk of malignant transformation [14,15].

Currently, no medicinal therapy exists for CeD, but today the gluten-induced pathogenic immunologic axis is harnessed for therapeutic design and development [16,17].

### Conventional CeD diagnosis, evaluation, and limitations

The golden standard for CeD diagnosis has been the gluten-dependent characteristic small intestinal mucosal lesion, villous atrophy with focus on existing crypt hyperplasia [18,19]. In clinics, the diagnosis is based on the worst injured mucosal finding in four distal duodenal biopsies, recommended to be reported according to the qualitative Marsh-Oberhuber classification, i.e. M-O 0/1, 2, 3a, 3b, and 3c [20].

Quantitative morphometric evaluation of CeD biopsies has been in use for decades in academic research. Here, the mucosal morphology is measured as a global mean of villous height and crypt depth ratios (VH:CrD) and separately the inflammation as density of CD3+ IELs, cells per 100 epithelial cells or cells per millimeter epithelium. In clinical drug trials, the mucosal injury continuum and change is mostly evaluated morphometrically [12,21–27].

Today, CeD diagnosis can accurately be established in part of patients without duodenal biopsy both in children and adults [28,29]. In children, biopsy remains an important diagnostic tool despite the widespread adoption of ESPGHAN non-biopsy criteria [28], and mucosal healing on a gluten-free diet generally follows the same pattern as in adults, often occurring more rapidly [30]. In therapeutic trials again, follow-up biopsies are likely to increase once therapeutics are approved, to determine eligibility. Histology remains the current reference standard but suffers from limited reproducibility and sensitivity as we discuss below.

### Emergence of transcriptomics

Recent advances in transcriptomic technologies have enabled comprehensive characterization of gene expression within the intestinal mucosa, providing unprecedented insight into the molecular landscape of CeD. These approaches have revealed disease-specific epithelial and immune activation signatures that are not captured by conventional histology [31–38] (Table 1). In addition, transcriptomic approaches markedly expand the dimensionality of mucosal evaluation by incorporating a broad range of molecular parameters, thereby moving beyond the limited set of traditional histological metrics such as VH:CrD and density of IELs. This enables a more comprehensive and biologically informative characterization of tissue state, capturing both structural and functional aspects of disease activity. Most importantly, molecular histomorphometry reading of CeD biopsies provides an objective readout as compared to traditional histopathology evaluation, both the qualitative M-O classification and quantitative morphometry.

**Table 1.** Key transcriptomic studies in CeD.

Study	Samples	Method	Context	Key findings	Comparison to histology	Key contribution
Bragde et al. 2018 [31]	Duodenal biopsies	RNA-seq	Active CeD	Immune, barrier and metabolic signatures	Correlate with Marsh scores	Detects early transcriptomic changes in pediatric patients
Leonard et al. 2019 [32]	Duodenal biopsies	RNA-seq	Active + treated	Immune, metabolic and spliceosome-related pathways	Correlate with Marsh scores and distinguish active vs. treated CeD	Expanded disease mechanisms
Loberman-Nachum et al. 2019 [33]	FFPE Duodenal biopsies	RNA-seq	Active CeD + Chron disease	Overlap with Crohn disease (immune/metabolic); proliferation increased in CeD	Supports diagnosis beyond histology	Clinical feasibility
Dotsenko et al. 2021 [35]	PaxFPE Biopsies GFD + challenge	RNA-seq	Gluten challenge	Gluten-induced transcriptomic changes. Persistent abnormalities during GFD	Predicts mucosal damage	Molecular histomorphometry tool
Dotsenko et al. 2024 [36]	PaxFPE Biopsies GFD + challenge and ZED1227-treated	RNA-seq	Gluten challenge TG2 inhibitor trial	Tracks treatment response. HLA-genotype tiering.	Captured changes beyond histology	Trial endpoint validation
Torinsson Naluai et al. 2025 [34]	Biopsies + blood	RNA-seq	Active, potential and treated CeD	Crostalk between antioxidant defense, immune regulation and metabolism	Distinguish CeD mucosa from healthy mucosa	Systemic disease insights in pediatric patients
Oliver et al. 2024 [37]	Intestinal samples	scRNA-seq	12 different diseases including CeD	Metaplastic epithelial cells in CeD	Cell-specific changes	High-resolution mapping
Richards et al. 2025 [38]	Duodenal biopsies	scRNA-seq	Active CeD	Stromal changes in CeD	Enterocyte loss correlates with Marsh score	Immune-stromal crosstalk

Although most transcriptomic studies have been conducted in adults, the molecular mechanisms underlying celiac enteropathy are considered similar across age groups. While transcriptome-wide studies integrated with quantitative morphometry are still lacking in pediatric cohorts, available paediatric transcriptomic studies support the expectation that molecular histomorphometry approaches will be applicable also in children [31,34].

### Aim of the review

The aim of this review is to synthesize current knowledge on molecular histomorphometry and the application of transcriptomic profiling in CeD. We focus on how these approaches improve understanding of mucosal pathology, address key limitations of traditional histological assessment, and provide novel, quantitative endpoints for diagnostic and therapeutic applications. In addition, we outline practical considerations and general principles for implementing transcriptomic-based analyses in both research and clinical settings.

## Discussion

1. **Limitations of Conventional Histology in Celiac Disease**

Traditional histological assessment in CeD lies in evaluating the architecture of the small intestine and the inflammation, today mostly using the M-O grouped classes [39]. The European Society for the Study of Coeliac Disease (ESsCD) guidelines recently recommended the M-O injury grouping to remain the reference measuring standard in CeD [20]. However, interobserver reproducibility in reading CeD histopathology using M-O classes 0/1, 2, 3a, 3b, and 3c showed unacceptable Kappa values being at best at 0.486 [40–42]. The study of Picarelli et al. stressed the limits of histological interpretation to be due to the lack of uniformity in using this qualitative grouping [43].

Misreadings occurred also in more recent studies, where reproducibility was far from optimal; focus being on villous atrophy and blunting without demanding crypts to be cut longitudinally, the hallmark for correct orientation [44–48]. There seem to be major differences in how to handle, process and evaluate biopsies for correct reading. In fact, at the Food and Drug Administration–sponsored 6th Gastroenterology Regulatory Endpoints and Advancement of Therapeutics Workshop there was consensus on the importance of relying on well-oriented samples and a standardized process for histologic scoring [49].

When evaluating inflammation in CeD, the review by Lindfors et al. shows the sensitivity of increased density of CD3+ IELs in untreated CeD to be 82-89% and that in detecting mild enteropathy CeD it is 59-93%. This means M-O classes 2 to 3c, fulfilling the morphological criteria for untreated CeD may not always have an increased density of IELs, i.e. exceeding 25 cells/100 epithelial cells [50]. This means that severe gluten-dependent morphological injury but with normal density of IELs cannot be classified according to the qualitative M-O groups.

Potential CeD patients being serum autoantibody positive but lacking histological findings meeting the diagnostic criteria will not be prescribed a GFD. Three-dimensional micro-computed tomographic imaging revealed early mucosal remodelling in these patients [51]. In fact, these patients may have already a gluten-induced extraintestinal manifestation even at a mucosal state of M-O 0 or 1 [52].

Standard operating procedures for processing of duodenal biopsies and validation of interobserver and intraobserver morphometric analyses, separately for mucosal morphology (VH:CrD) and inflammation (IELs) in CeD have been reported [53]. In fact, quantitative histopathology measurements have extensively been used to assess CeD activity in clinical drug trials [12,21–27]. However, Al-Toma et al. stated that morphometric biopsy evaluation faces challenges in clinical adoption due to practical limitations, lack of standardisation, and insufficient evidence of superiority over existing M-O method [20].

Consequently, accurate assessment of mucosal injury and recovery remains essential for both clinical management and therapeutic development. While histology remains the cornerstone of disease assessment, both qualitative and quantitative histopathological interpretations are subjective, as they depend on the cutting angle of the biopsy and the orientation of the villus-crypt units. Transcriptomic approaches, in turn, offer a complementary and more objective insight into the molecular processes underlying mucosal injury, repair, and treatment response (Figure 1).

**Figure 1.**
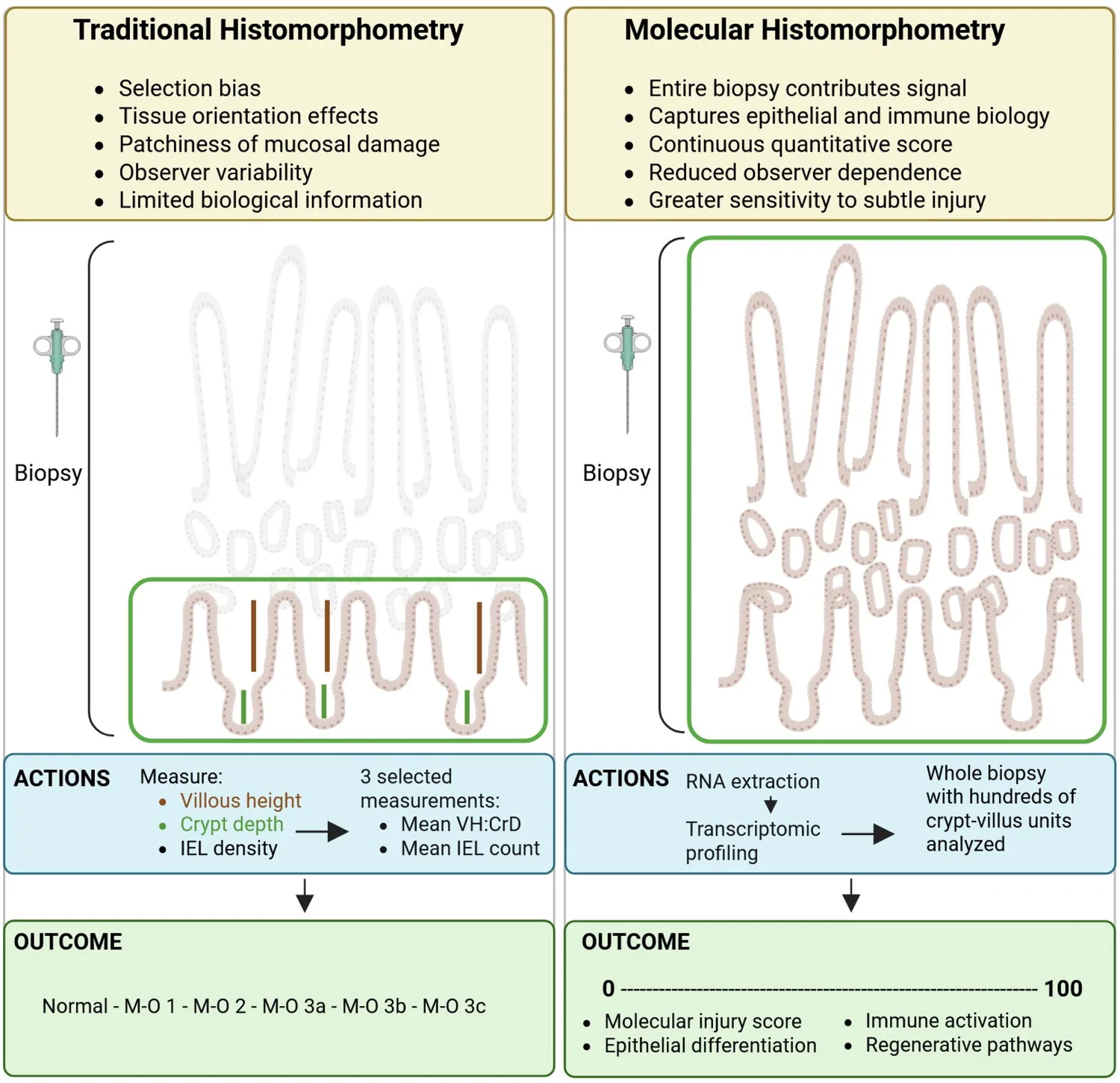
**Sampling-based histomorphometry versus whole-biopsy molecular histomorphometry in celiac disease.** Traditional histomorphometry relies on manual measurement of a limited number of selected crypt-villus units, typically assessing villous height, crypt depth, and IEL density. Consequently, results may be influenced by biopsy orientation, observer-dependent selection of measurement sites, and the patchy distribution of mucosal injury. In contrast, molecular histomorphometry derives a quantitative molecular injury score from transcriptomic profiling of the entire biopsy specimen. The resulting molecular signal integrates gene-expression information originating from hundreds of crypt-villus units and their associated immune and stromal compartments, thereby capturing epithelial differentiation, immune activation, and tissue remodelling across the whole tissue sample. Unlike conventional morphometry, which requires well-oriented longitudinally sectioned villi and crypts (brown vs. grey crypt-villus units) for reliable measurements, molecular histomorphometry incorporates transcriptomic information from the entire biopsy specimen, including cross-sectioned crypts and other tissue regions unsuitable for conventional morphometric evaluation. This approach provides an objective, continuous, and biologically informative measure of mucosal injury with increased sensitivity to subtle disease activity and treatment responses. Created with Biorender.com.

1. **Concept of Molecular Histomorphometry in Celiac Disease**

### From morphology to molecular quantification

Conventional histological assessment remains the cornerstone for evaluating mucosal injury in CeD, yet it provides only a semi-quantitative and largely descriptive view of tissue architecture (Figure 1). As a result, important biological changes underlying disease activity may remain undetected, particularly in treated patients or during early or subtle disease stages. These limitations have prompted a shift toward approaches that extend beyond morphology to enable quantitative assessment of tissue biology.

**Definition.** Molecular histomorphometry is a recently proposed approach that integrates gene expression profiling with conventional histological features to enable continuous, quantitative evaluation of mucosal structure and activity. Rather than relying on categorical classifications such as the M-O grouping, this approach captures disease severity along a continuum by linking transcriptional programs to morphological alteration. Importantly, it does not replace histology, but refines and complements it by providing objective, reproducible, and biologically informative readouts (Box 1).

Box 1. Context from Other Fields: Integration of Molecular and Histological Tissue Assessment

Integration of molecular data with tissue morphology is increasingly recognized as a powerful approach for characterizing disease across multiple biomedical fields. Spatial transcriptomics has enabled direct mapping of gene expression within intact tissue architecture, revealing cellular heterogeneity and molecular gradients that are not discernible by conventional histology alone [54].

In oncology, multimodal approaches combining histopathology with transcriptomic and proteogenomic data have improved tumour classification and enabled identification of biologically meaningful subtypes linked to clinical outcomes. Moreover, computational pathology methods now allow prediction of gene expression profiles directly from histological images, further demonstrating the strong coupling between tissue structure and molecular activity [55,56].

More broadly, integrative frameworks combining clinical, molecular, and histopathological information—sometimes termed ‘tissunomics’—highlight a paradigm shift towards multidimensional tissue characterization. Together, these developments underscore a transition from descriptive morphology toward quantitative, molecularly informed tissue assessment [57].

### Biological rationale: the intestinal mucosa as an ideal system

The small intestinal mucosa represents a uniquely suitable tissue for this approach. Along the crypt-villus axis, epithelial cells undergo tightly regulated processes of proliferation, differentiation, and maturation that are reflected in pronounced gradients of gene expression [60] (Figure 2). This spatial organization creates a strong coupling between tissue architecture and underlying transcriptional programs. In CeD, this highly ordered system is disrupted, with crypt hyperplasia, villous atrophy, and immune activation accompanied by coordinated shifts in epithelial and immune gene expression. The rapid regenerative capacity and continuous turnover of the epithelium (renewal cycle is 3–5 days and up to 10^11^ epithelial cells are lost every day in humans [59] further amplify these molecular signals, enabling sensitive detection of dynamic changes in disease activity. This spatial organization is governed by canonical Wnt/β-catenin signalling, which regulates the balance between crypt stem cell maintenance and villus differentiation. Modulation of Wnt activity in intestinal organoids reveals approximately 2,600 genes differentially expressed along the crypt–villus axis, highlighting the extent of transcriptional gradients underlying mucosal structure and supporting the intestine as an ideal system for molecular histomorphometry. Comparison with experimentally defined Wnt-responsive genes revealed evidence of hyperactive Wnt signalling in CeD. Approximately one-quarter of genes altered by gluten challenge (24.6%) and a similar proportion of genes differentially expressed in patients on a gluten-free diet (28.2%) were consistent with increased Wnt activity, suggesting that disruption of the Wnt-regulated crypt–villus differentiation program is a major component of both active and treated CeD [58].

**Figure 2.**
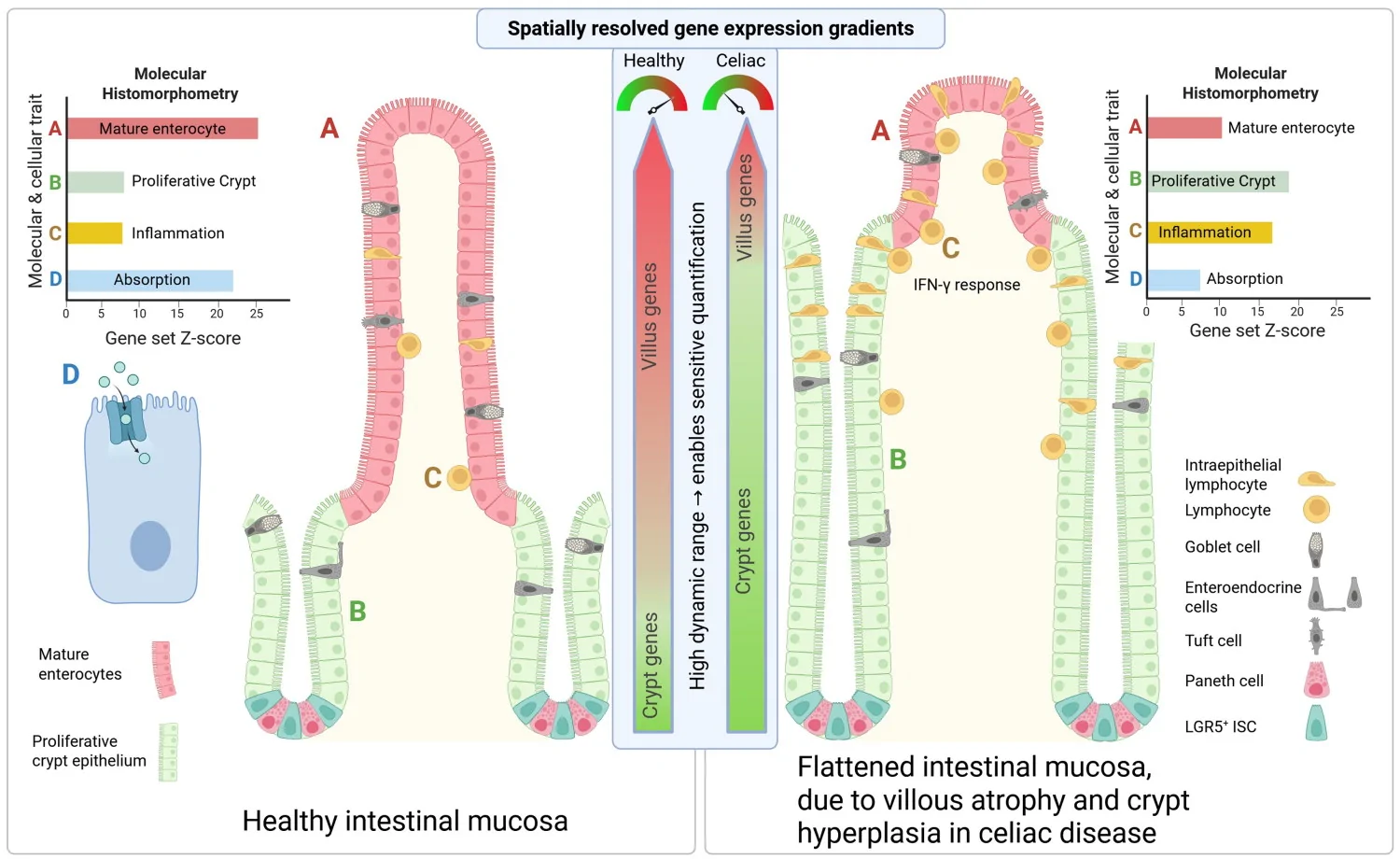
**The intestinal crypt–villus axis as a model for molecular histomorphometry.** Spatially organized epithelial compartments exhibit strong gene expression gradients corresponding to proliferation (crypt) and differentiation (villus) with more than 2600 genes differentially expressed [58]. In CeD, this architecture and its associated transcriptional programs are disrupted, enabling sensitive molecular quantification of mucosal injury. Created with Biorender.com.

### Evidence from transcriptomic studies

Evidence from transcriptomic studies strongly supports this conceptual framework. Early bulk transcriptomic analyses established the presence of disease-specific epithelial and immune activation signatures and demonstrated that gene expression profiles can distinguish CeD from controls. Subsequent RNA sequencing studies extended these findings by identifying novel immune, metabolic, and epithelial pathways and by demonstrating feasibility in routine diagnostic samples [32,33].

Importantly, gluten challenge and interventional studies have provided dynamic evidence of disease activity: transcriptomic profiling reveals persistent molecular abnormalities despite histological normalization and sensitively captures gluten-induced injury as well as therapeutic responses [35,36].

More recently, single-cell transcriptomic approaches have provided high-resolution insight into epithelial remodelling, stem cell expansion, and stromal–immune interactions [37,38], directly linking transcriptional programs to the structural alterations characteristic of mucosal pathology.

Together, these studies demonstrate that transcriptomic readouts not only complement histological findings but provide a more sensitive, quantitative, and mechanistically informative assessment of disease state (Table 1).

1. **Transcriptomic Profiling of the Celiac Mucosa**

### Active disease signatures

Transcriptomic analyses of active CeD reveal a coordinated molecular program linking immune activation and epithelial remodelling. A dominant feature is interferon-γ–driven signaling, reflecting robust adaptive immune activation within the intestinal mucosa [32–38,61]. A substantial proportion, about half, of gluten-induced gene expression changes are associated with interferon pathways, highlighting their central role in disease pathogenesis [36]. These immune responses are accompanied by upregulation of antigen presentation, cytokine signalling, and cytotoxic effector mechanisms, collectively contributing to epithelial injury.

In parallel, the epithelial compartment exhibits marked disruption of differentiation programs. Gene expression profiling demonstrates suppression of mature enterocyte functions together with a shift towards immature, crypt-like transcriptional states, indicative of epithelial stress and impaired maturation. This loss of differentiation contributes directly to the structural and functional changes characteristic of villous atrophy.

Active disease is further characterized by activation of regenerative pathways, including Wnt signalling and proliferation-associated gene programs [35,58]. These transcriptional changes reflect expansion of proliferative crypt cell populations and increased epithelial turnover, corresponding to crypt hyperplasia observed histologically. Together, these pathways define an integrated epithelial–immune–regenerative response that underlies mucosal injury in CeD.

### Transcriptomics in treated celiac disease (gluten-free diet)

In patients adhering to a gluten-free diet, transcriptomic profiling demonstrates that mucosal recovery is often incomplete at the molecular level [35]. Despite normalization of histological indices, persistent alterations in immune and epithelial gene expression are frequently observed. In particular, genes related to micronutrient absorption remain dysregulated compared with healthy controls, indicating ongoing subclinical disease activity which probably explains reported clinical micronutrient deficiencies in patients following a strict GFD [62–65]. These findings highlight that histological remission does not necessarily correspond to full biological recovery.

### Gluten challenge responses

Gluten challenge studies reveal that transcriptional responses to gluten exposure are rapid and highly sensitive. Following gluten ingestion, coordinated activation of immune pathways and reversion to immature epithelial gene programs can be detected [35,36]. The magnitude of these transcriptional changes correlates closely with the degree of mucosal injury, supporting their use as quantitative markers of disease activity.

1. **Molecular Histomorphometry in Practice**

### Transcriptomic prediction of mucosal injury

The close relationship between gene expression and tissue architecture enables the construction of molecular models that quantitatively estimate mucosal injury. Transcript levels correlate strongly with both villous architecture and mucosal inflammation. In a proof-of-concept study, models based on only four transcripts explained 97.2% of the variability in VH:CrD and 98.0% of the variability in the density of CD3+ IELs. Notably, transcriptome-wide data could be reduced to small predictive gene sets. For example, ATP8B2, PLA2R1, PDIA3, and TM4SF4 predicted villous architecture, whereas CCL5, AC004067.6, PDIA3, and SLC15A1 predicted inflammatory activity [35] (Figure 3). These findings suggest that a limited number of biologically informative transcripts may capture the major structural and inflammatory features of CeD traditionally assessed by histology. Importantly, these findings have subsequently been supported by independent transcriptomic studies, including those of Dotsenko et al. (2021) [35] and Dotsenko et al. (2024) [36], which demonstrated the reproducibility and utility of molecular histomorphometry in distinct clinical settings. Nevertheless, further validation in larger, independent, and geographically diverse cohorts is required before these approaches can be considered broadly applicable clinical tools.

**Figure 3.**
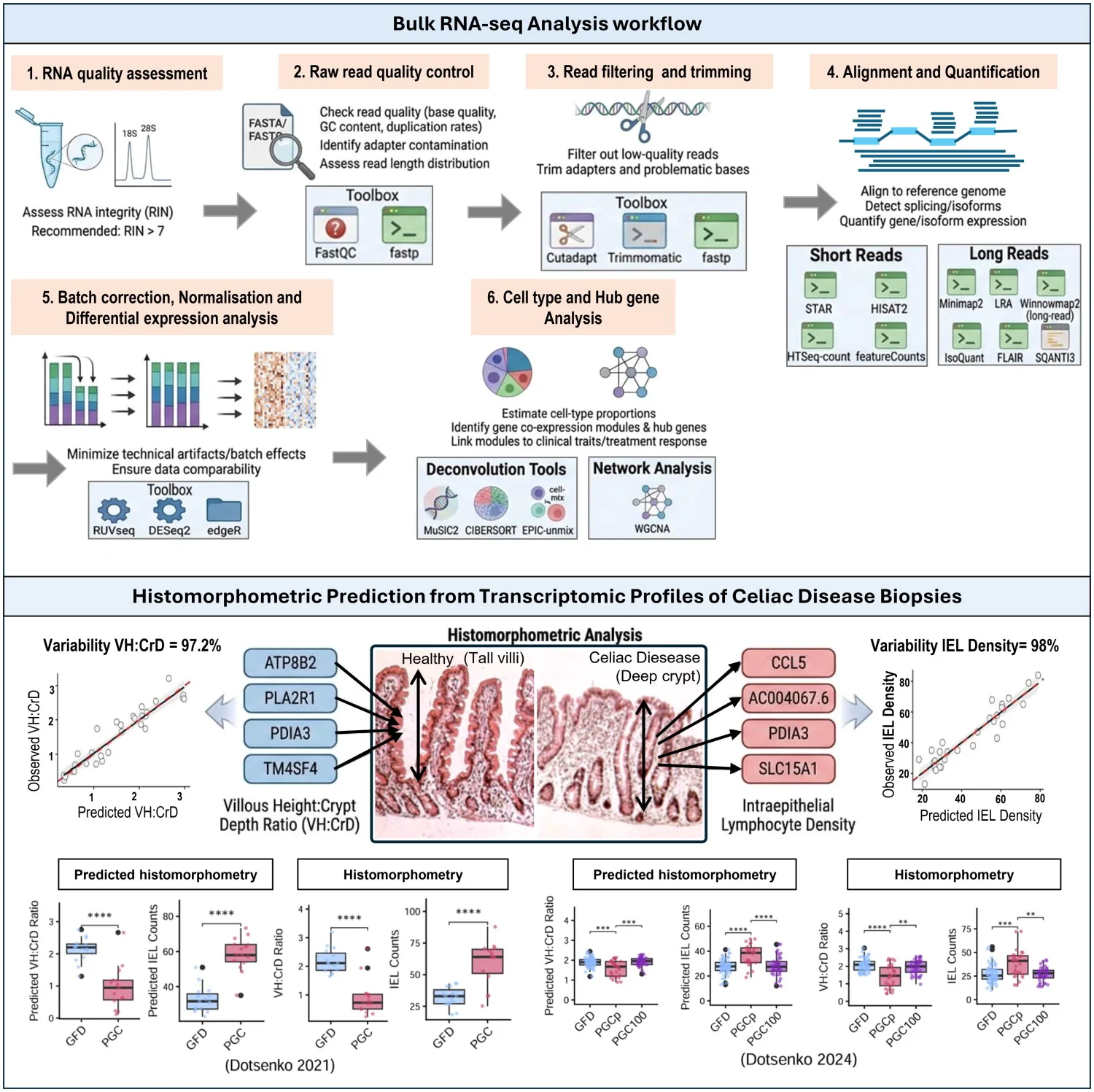
**Transcriptomic workflow and molecular histomorphometry in celiac disease.** Upper panel: overview of a typical bulk RNA-sequencing workflow, including RNA quality assessment, read processing, alignment, quantification, normalization, and downstream analyses for cellular composition, gene networks, and biomarker discovery. Lower panel: development of molecular histomorphometry models from transcriptomic data. Expression of selected genes predicts conventional histological measures, including villous height-to-crypt depth ratio (VH:CrD) and intraepithelial lymphocyte (IEL) density, enabling quantitative molecular assessment of mucosal injury. Molecular predictions closely mirror conventional histomorphometric measurements and capture treatment-associated changes in disease activity (data adopted from Dotsenko et al. 2021 [35] and 2024[36]). See main text for methodological considerations and analytical recommendations. GFD, Gluten-free diet; PGCp, Post-gluten challenge patient treated with placebo; PGC100, Post-gluten challenge patient treated with 100mg of ZED1227 drug. Created with Biorender.com.

### Towards targeted molecular panels

Although transcriptome-wide sequencing has been instrumental in developing molecular histomorphometry, routine clinical implementation will likely rely on focused gene panels. Candidate markers may represent complementary aspects of disease biology, including epithelial differentiation (APOA4), proliferation (MKI67), immune activation (CCL5) and tissue remodelling. In particular, the APOA4:MKI67 ratio has been proposed as a marker reflecting the balance between differentiated villus epithelium and proliferative crypt compartments and may serve as a simple molecular surrogate of crypt–villus architecture [66,67].

### Assessing treatment efficacy in therapeutic trials

Molecular histomorphometry has the potential to enable quantitative assessment of therapeutic responses by measuring transcriptomic changes associated with epithelial differentiation, immune activation, and tissue remodelling. In gluten challenge studies, gene expression changes closely correlate with the extent of mucosal injury and inflammation, supporting their use as continuous measures of treatment response. The clinical utility of this approach was demonstrated in the phase 2 trial of the TG2 inhibitor ZED1227. Transcriptomic analyses showed that treatment prevented gluten-induced molecular alterations associated with inflammation, epithelial differentiation, nutrient absorption, and mucosal morphology, maintaining a transcriptional profile resembling that observed during a GFD [36]. Notably, nearly half of the gluten-responsive gene expression changes were linked to epithelial interferon-γ responses, highlighting both a major disease pathway and a sensitive molecular marker of therapeutic efficacy. Recent multi-omics analyses further showed that ZED1227 normalized gluten-induced systemic lipidomic, proteomic, and DNA methylation changes, extending molecular treatment effects beyond the intestinal mucosa [68].

### Towards personalized treatment strategies

As it has been shown that gene dose of HLA-DQ2/DQ8 influence the severity of CeD [69,70], thus beyond serving as a treatment endpoint, transcriptomic profiling may facilitate precision medicine approaches in CeD. In the ZED1227 study, molecular responses differed according to HLA-DQ2/DQ8 gene dosage [36], suggesting that molecular response-guided HLA-based stratification could help identify patients most likely to benefit from targeted interventions and optimize therapeutic responses.

1. **Practical pipeline and guidelines**

### Platform selection: targeted panels versus RNA sequencing

For understanding disease states, a targeted gene panel comprising genes associated with histomorphometric prediction and disease-specific molecular signatures, together with HLA susceptibility markers, can be cost-effective, time-efficient, personalized, and highly informative. A particularly attractive example is the APOA4:MKI67 ratio, which reflects the balance between mature villus enterocytes and proliferating crypt cells and therefore serves as a molecular representation of crypt–villus morphology [66,67].

However, for biomarker discovery and exploratory analyses, RNA sequencing (RNA-seq) remains the preferred approach. The choice of RNA-seq methodology depends on the specific biological question being addressed. If the focus is on protein-coding gene expression, poly(A)-selected RNA-seq is generally the most appropriate option. In contrast, when investigating regulatory RNA species, such as microRNAs (miRNAs), long non-coding RNAs (lncRNAs), and small nucleolar RNAs (snoRNAs), total RNA-seq provides broader transcriptome coverage. Furthermore, for studies aimed at characterizing transcript isoforms, alternative splicing events, and transcript structural variation, long-read RNA sequencing technologies offer significant advantages over conventional short-read approaches.

In addition to analytical performance, reproducibility and clinical scalability should be carefully considered when selecting a transcriptomic approach. Methods intended for clinical implementation should demonstrate robust reproducibility across laboratories, platforms, and sample types, while also being cost-effective, standardized, and scalable for routine diagnostic and translational applications.

### RNA quality and pre-processing

The interpretation of sequencing data is highly dependent on the quality control (QC) steps applied throughout the data processing workflow (Figure 3). At the initial stage, RNA integrity is critical, as higher-quality RNA improves downstream reproducibility; typically, an RNA integrity number (RIN) >7 is recommended for both targeted panel screening and RNA-seq applications. RNA-seq analysis begins with rigorous quality assessment of raw reads, including filtering based on base quality scores, overall read quality, adapter contamination, GC content, sequence duplication, and read length distribution, which may vary depending on library preparation. These preprocessing steps can be efficiently performed using widely used tools such as FastQC [71], fastp [72], Cutadapt [73] and Trimmomatic [74].

### Alignment and quantification

Subsequent alignment and quantification require careful consideration of reference genome versions, index files, and parameter settings, as these can introduce variability across pipelines. A range of established tools is available depending on the experimental design. For general short-read mapping and splice-aware alignment, STAR [75] is widely used and capable of detecting splicing events and isoforms. Alternatively, HISAT2 [76] combined with HTSeq-count [77] or featureCounts [78] offers a faster and more resource-efficient approach for gene-level quantification. For long-read data, aligners such as Minimap2 [79], LRA [80], Winnowmap2 [81], and STAR (in long-read settings) are commonly used, while downstream isoform-level quantification tools include IsoQuant [82], FLAIR [83] and SQANTI3 [84].

### Normalization, batch correction, and pipeline standardization

To minimize pipeline-related variability and enhance reproducibility, the use of standardized, containerized workflows is strongly recommended. Frameworks such as nf-core provide well-documented, portable, and reproducible pipelines that can be deployed across diverse computational environments. To address batch effects and technical biases arising from different platforms, normalization and batch correction methods implemented in tools such as RUVseq [85], DESeq2 [86] and edgeR [87] are commonly applied during downstream analysis. Collectively, these standardized workflows support robust data processing and facilitate the integration of heterogeneous bulk RNA-seq datasets.

### Exploratory analysis in the context of clinical trials

Beyond routine differential expression analysis, bulk RNA-seq data can be leveraged to infer changes in cellular composition between drug-treated and control groups. Computational deconvolution methods such as MuSiC2 [88] and CIBERSORT [89] utilize publicly available single-cell RNA-seq (scRNA-seq) or single-nucleus RNA-seq (snRNA-seq) reference datasets to estimate cell type proportions within bulk samples. This provides a cost-effective alternative to large-scale single-cell profiling while enabling analysis across larger clinical cohorts.

More recently, Bayesian framework–based deconvolution tool, EPIC-unmix [90] has enabled estimation of cell type-specific gene expression profiles directly from bulk RNA-seq data. These approaches allow for the resolution of transcriptional changes at the level of individual cell types, offering deeper insight into treatment effects, and supporting the identification of potential therapeutic targets and biomarkers.

In addition, Weighted Gene Co-expression Network Analysis (WGCNA) [91] provides a complementary systems biology approach for large clinical datasets. Rather than focusing on individual differentially expressed genes, WGCNA constructs gene co-expression networks to identify modules of highly correlated genes. These modules can then be associated with clinical traits or experimental conditions to reveal biologically meaningful relationships. Within key modules, hub genes due to their central network connectivity are often prioritized as candidate biomarkers or therapeutic targets.

1. **Challenges and limitations of molecular histomorphometry**

Despite its considerable promise, molecular histomorphometry faces several challenges that must be addressed before widespread clinical implementation. First, the approach still requires an intestinal biopsy and therefore does not eliminate the need for endoscopic tissue sampling. In addition, transcriptomic analyses are sensitive to RNA quality, sample handling, storage conditions, and technical variation introduced during sequencing and data processing.

Further sources of variability include differences between analytical platforms, library preparation methods, and bioinformatics pipelines, which may affect reproducibility across studies and centers. At present, there is also no universally accepted gene panel or molecular injury score for CeD, limiting comparability between investigations and hindering clinical standardization.

From a practical perspective, transcriptomic analyses remain more expensive and technically demanding than conventional histopathological assessments, although costs continue to decline. Regulatory acceptance will require extensive analytical and clinical validation, including demonstration of robustness, reproducibility, and clinical utility across independent cohorts and laboratories.

Importantly, molecular histomorphometry should currently be viewed as a complement rather than a replacement for traditional histology. Histological evaluation provides critical information on tissue architecture and remains the diagnostic reference standard in many clinical settings. Finally, the interpretation of molecular findings in borderline conditions, such as potential CeD or patients with minimal mucosal abnormalities, remains incompletely understood and requires further investigation.

Overall, continued standardization, multicenter validation, and regulatory qualification will be essential before molecular histomorphometry can be routinely incorporated into clinical practice and therapeutic trials.

## Conclusions

Histological assessment has served as the foundation of CeD diagnosis and therapeutic evaluation for decades, but it remains constrained by categorical scoring systems, observer variability, sampling limitations, and limited biological resolution. Advances in transcriptomics now enable quantitative characterization of mucosal injury at a molecular level, providing information that extends beyond conventional morphology.

Molecular histomorphometry represents a promising conceptual shift from evaluation of a small number of selected crypt-villus units toward integrated analysis of the entire biopsy specimen. By linking transcriptional programs with tissue architecture, this approach generates continuous measures of disease activity that reflect epithelial differentiation, immune activation, and tissue remodelling.

Although further standardization, multicentre validation, and regulatory qualification are required, accumulating evidence suggests that molecular endpoints can complement and, in selected applications, outperform traditional histological assessments. Their value may be particularly evident in therapeutic trials, where improved sensitivity could facilitate patient stratification, endpoint assessment, and detection of treatment responses.

Looking forward, targeted transcriptomic panels, integration with digital pathology and artificial intelligence, and emerging spatial and single-cell technologies are likely to further enhance molecular assessment of CeD. More broadly, molecular histomorphometry exemplifies the ongoing transition from descriptive tissue pathology toward quantitative molecular pathology. As these technologies mature, they may transform CeD diagnostics, therapeutic development, and precision medicine, shifting disease assessment from categorical histological classifications toward direct measurement of underlying disease biology.

## Data Availability

There is no data associated with this research.

## References

[1] Mäki M, Collin P. Coeliac disease. Lancet. 1997;349(9067):1755–1759. doi: 10.1016/S0140-6736(96)70237-4. 9193393

[2] Murray JA, Husby S. Celiac disease. N Engl J Med. 2026;394(14):1421–1429. doi: 10.1056/NEJMcp2415548. 41950475

[3] Abadie V, Han AS, Jabri B, et al. New insights on genes, gluten, and immunopathogenesis of celiac disease. Gastroenterology. 2024;167(1):4–22. doi: 10.1053/j.gastro.2024.03.042. PMC11283582 38670280

[4] Singh P, Arora A, Strand TA, et al. Global prevalence of celiac disease: systematic review and meta-analysis. Clin Gastroenterol Hepatol. 2018;16(6):823–836.e2. doi: 10.1016/j.cgh.2017.06.037. 29551598

[5] Mustalahti K, Catassi C, Reunanen A, et al. The prevalence of celiac disease in Europe: results of a centralized, international mass screening project. Ann Med. 2010;42(8):587–595. doi: 10.3109/07853890.2010.505931. 21070098

[6] Lohi S, Mustalahti K, Kaukinen K, et al. Increasing prevalence of coeliac disease over time. Aliment Pharmacol Ther. 2007;26(9):1217–1225. doi: 10.1111/j.1365-2036.2007.03502.x. 17944736

[7] Gibson PR. Commentary: recognising the boom in coeliac disease prevalence was more than just increased awareness. Aliment Pharmacol Ther. 2020;51(1):207–208. doi: 10.1111/apt.15552. 31850552

[8] Taavela J, Kurppa K, Jääskeläinen T, et al. Trends in the prevalence rates and predictive factors of coeliac disease: a long-term nationwide follow-up study. Aliment Pharmacol Ther. 2024;59(3):372–379. doi: 10.1111/apt.17803. 37946663

[9] Kaukinen K, Collin P, Mäki M. [Celiac disease–a diagnostic and therapeutic challenge]. Duodecim. 2010;126(3):245–254. 20402035

[10] Dieterich W, Ehnis T, Bauer M, et al. Identification of tissue transglutaminase as the autoantigen of celiac disease. Nat Med. 1997;3(7):797–801. doi: 10.1038/nm0797-797. 9212111

[11] Mehta S, Chauhan A, Vignesh D, et al. Global prevalence of adherence to gluten-free diet in patients with celiac disease: a systematic review and meta-analysis. Am J Gastroenterol. 2026; Published online March 3,. doi: 10.14309/ajg.0000000000003985. 41773976

[12] Daveson AJM, Popp A, Taavela J, et al. Baseline quantitative histology in therapeutics trials reveals villus atrophy in most patients with coeliac disease who appear well controlled on gluten-free diet. GastroHep. 2020;2(1):22–30. Published online January 20, ygh2. doi: 10.1002/ygh2.380.

[13] Guennouni M, Admou B, Khoudri NE, et al. Gluten contamination in labelled gluten-free, naturally gluten-free and meals in food services in low-, middle- and high-income countries: a systematic review and meta-analysis. Br J Nutr. 2022;127(10):1528–1542. doi: 10.1017/S0007114521002488. 34753529

[14] Ilus T, Kaukinen K, Virta LJ, et al. Refractory coeliac disease in a country with a high prevalence of clinically-diagnosed coeliac disease. Aliment Pharmacol Ther. 2014;39(4):418–425. doi: 10.1111/apt.12606. 24387637

[15] Al-Toma A, Branchi F, Zingone F, et al. European Society for the Study of Coeliac Disease (ESsCD) 2025 Updated Guidelines on the Diagnosis and Management of Coeliac Disease in Adults. Part 2: management, Follow-Up, and Complex Disease Courses. United European Gastroenterol J. 2026;14(2):e70195. doi: 10.1002/ueg2.70195. PMC13097674 41831197

[16] Besser HA, Khosla C. Celiac disease: mechanisms and emerging therapeutics. Trends Pharmacol Sci. 2023;44(12):949–962. doi: 10.1016/j.tips.2023.09.006. PMC10843302 37839914

[17] Taavela J, Elli L, Bouma G, et al. Upcoming treatments in celiac disease: from luminal enzymes to oral immune tolerance. United European Gastroenterol J. 2026;14(3):e70222. doi: 10.1002/ueg2.70222. PMC13109976 42033586

[18] Rubin CE, Brandborg LL, Phelps PC, et al. Studies of celiac disease. I. The apparent identical and specific nature of the duodenal and proximal jejunal lesion in celiac disease and idiopathic sprue. Gastroenterology. 1960;38(1):28–IN1. doi: 10.1016/S0016-5085(60)80115-1. 14439871

[19] Thurlbeck WM, Benson JA, Dudley HR. The histopathologic changes of sprue and their significance. Am J Clin Pathol. 1960;34:108–117. doi: 10.1093/ajcp/34.2.108. 13776777

[20] Al-Toma A, Zingone F, Branchi F, et al. European Society for the Study of Coeliac Disease 2025 Updated Guidelines on the Diagnosis and Management of Coeliac Disease in Adults. Part 1: diagnostic Approach. United European Gastroenterol J. 2025;13(10):1855–1886. doi: 10.1002/ueg2.70119. PMC12704582 40999951

[21] Lähdeaho ML, Mäki M, Laurila K, et al. Small- bowel mucosal changes and antibody responses after low- and moderate-dose gluten challenge in celiac disease. BMC Gastroenterol. 2011;11:129. doi: 10.1186/1471-230X-11-129. PMC3240817 22115041

[22] Lähdeaho ML, Kaukinen K, Laurila K, et al. Glutenase ALV003 attenuates gluten-induced mucosal injury in patients with celiac disease. Gastroenterology. 2014;146(7):1649–1658. doi: 10.1053/j.gastro.2014.02.031. 24583059

[23] Goel G, King T, Daveson AJ, et al. Epitope-specific immunotherapy targeting CD4-positive T cells in coeliac disease: two randomised, double-blind, placebo-controlled phase 1 studies. Lancet Gastroenterol Hepatol. 2017;2(7):479–493. doi: 10.1016/S2468-1253(17)30110-3. PMC5676538 28506538

[24] Murray JA, Kelly CP, Green PHR, et al. No difference between latiglutenase and placebo in reducing villous atrophy or improving symptoms in patients with symptomatic celiac disease. Gastroenterology. 2017;152(4):787–798.e2. doi: 10.1053/j.gastro.2016.11.004. 27864127

[25] Lähdeaho ML, Scheinin M, Vuotikka P, et al. Safety and efficacy of AMG 714 in adults with coeliac disease exposed to gluten challenge: a phase 2a, randomised, double-blind, placebo-controlled study. Lancet Gastroenterol Hepatol. 2019;4(12):948–959. doi: 10.1016/S2468-1253(19)30264-X. 31494096

[26] Schuppan D, Mäki M, Lundin KEA, et al. A randomized trial of a transglutaminase 2 inhibitor for celiac disease. N Engl J Med. 2021;385(1):35–45. doi: 10.1056/NEJMoa2032441. 34192430

[27] Murray JA, Syage JA, Wu TT, et al. Latiglutenase protects the mucosa and attenuates symptom severity in patients with celiac disease exposed to a gluten challenge. Gastroenterology. 2022;163(6):1510–1521.e6. doi: 10.1053/j.gastro.2022.07.071. PMC9707643 35931103

[28] Husby S, Koletzko S, Korponay-Szabó I, et al. European Society Paediatric Gastroenterology, Hepatology and Nutrition Guidelines for Diagnosing Coeliac Disease 2020. J Pediatr Gastroenterol Nutr. 2020;70(1):141–156. doi: 10.1097/MPG.0000000000002497. 31568151

[29] Shiha MG, Nandi N, Raju SA, et al. Accuracy of the no-biopsy approach for the diagnosis of celiac disease in adults: a systematic review and meta-analysis. Gastroenterology. 2024;166(4):620–630. doi: 10.1053/j.gastro.2023.12.023. 38176661

[30] Kuitunen P, Kosnai I, Savilahti E. Morphometric study of the jejunal mucosa in various childhood enteropathies with special reference to intraepithelial lymphocytes. J Pediatr Gastroenterol Nutr. 1982;1(4):525–531. doi: 10.1097/00005176-198212000-00012. 7186067

[31] Bragde H, Jansson U, Fredrikson M, et al. Celiac disease biomarkers identified by transcriptome analysis of small intestinal biopsies. Cell Mol Life Sci. 2018;75(23):4385–4401. doi: 10.1007/s00018-018-2898-5. PMC6208765 30097691

[32] Leonard MM, Bai Y, Serena G, et al. RNA sequencing of intestinal mucosa reveals novel pathways functionally linked to celiac disease pathogenesis. Sestak K, ed. PLoS One. 2019;14(4):e0215132. doi: 10.1371/journal.pone.0215132. PMC6472737 30998704

[33] Loberman-Nachum N, Sosnovski K, Di Segni A, et al. Defining the celiac disease transcriptome using clinical pathology specimens reveals biologic pathways and supports diagnosis. Sci Rep. 2019;9(1):16163. doi: 10.1038/s41598-019-52733-1. PMC6838157 31700112

[34] Torinsson Naluai Å, Sabbag S, Abrahamsson S, et al. Whole genome transcriptional analysis of intestinal biopsies and blood cells indicate genes involved in antioxidant defense systems, amino acid metabolism and antigen presentation in the pathogenesis of celiac disease. BMC Med. 2025;23(1):507. doi: 10.1186/s12916-025-04261-1. PMC12398127 40883722

[35] Dotsenko V, Oittinen M, Taavela J, et al. Genome-wide transcriptomic analysis of intestinal mucosa in celiac disease patients on a gluten-free diet and postgluten challenge. Cell Mol Gastroenterol Hepatol. 2021;11(1):13–32. doi: 10.1016/j.jcmgh.2020.07.010. PMC7593586 32745639

[36] Dotsenko V, Tewes B, Hils M, et al. Transcriptomic analysis of intestine following administration of a transglutaminase 2 inhibitor to prevent gluten-induced intestinal damage in celiac disease. Nat Immunol. 2024;25(7):1218–1230. doi: 10.1038/s41590-024-01867-0. PMC11224021 38914866

[37] Oliver AJ, Huang N, Bartolome-Casado R, et al. Single-cell integration reveals metaplasia in inflammatory gut diseases. Nature. 2024;635(8039):699–707. doi: 10.1038/s41586-024-07571-1. PMC11578898 39567783

[38] Richards D, Sohn K, Basavarajappa SC, et al. Immune signaling mediates stromal changes to support epithelial reprogramming in celiac duodenum. Cell Rep. 2025;44(8):116039. doi: 10.1016/j.celrep.2025.116039. 40694474

[39] Oberhuber G, Granditsch G, Vogelsang H. The histopathology of coeliac disease: time for a standardized report scheme for pathologists. Eur J Gastroenterol Hepatol. 1999;11(10):1185–1194. doi: 10.1097/00042737-199910000-00019. 10524652

[40] Corazza GR, Villanacci V, Zambelli C, et al. Comparison of the interobserver reproducibility with different histologic criteria used in celiac disease. Clin Gastroenterol Hepatol. 2007;5(7):838–843. doi: 10.1016/j.cgh.2007.03.019. 17544877

[41] Mubarak A, Nikkels P, Houwen R, et al. Reproducibility of the histological diagnosis of celiac disease. Scand J Gastroenterol. 2011;46(9):1065–1073. doi: 10.3109/00365521.2011.589471. 21668407

[42] Arguelles-Grande C, Tennyson CA, Lewis SK, et al. Variability in small bowel histopathology reporting between different pathology practice settings: impact on the diagnosis of coeliac disease. J Clin Pathol. 2012;65(3):242–247. doi: 10.1136/jclinpath-2011-200372. 22081783

[43] Picarelli A, Borghini R, Donato G, et al. Weaknesses of histological analysis in celiac disease diagnosis: new possible scenarios. Scand J Gastroenterol. 2014;49(11):1318–1324. doi: 10.3109/00365521.2014.948052. 25113634

[44] Werkstetter KJ, Korponay-Szabó IR, Popp A, et al. Accuracy in diagnosis of celiac disease without biopsies in clinical practice. Gastroenterology. 2017;153(4):924–935. doi: 10.1053/j.gastro.2017.06.002. 28624578

[45] Villanacci V, Lorenzi L, Donato F, et al. Histopathological evaluation of duodenal biopsy in the PreventCD project. An observational interobserver agreement study. APMIS. 2018;126(3):208–214. doi: 10.1111/apm.12812. 29372596

[46] Maxwell JR. Comparison of inter-reader agreement for composite celiac histology endpoints and contrast with traditional histology endpoints. Accessed July 22, 2026. https://gutflix.eu/search/d/62661c22-9112-11ef-b058-0242ac140004.

[47] Robert ME, Crowe SE, Burgart L, et al. Statement on best practices in the use of pathology as a diagnostic tool for celiac disease: a guide for clinicians and pathologists. Am J Surg Pathol. 2018;42(9):e44–e58. doi: 10.1097/PAS.0000000000001107. 29923907

[48] Villanacci V, Del Sordo R, Casella G, et al. The correct methodological approach to the diagnosis of celiac disease: the point of view of the pathologist. Gastroenterol Hepatol Bed Bench. 2023;16(2):129–135. doi: 10.22037/ghfbb.v16i2.2704. PMC10404828 37554758

[49] Lavine I, Seo S, Tomaino J. Development of celiac disease therapeutics: the sixth gastroenterology regulatory endpoints and the advancement of therapeutics workshop. Gastro Hep Adv. 2023;2(3):294–297. doi: 10.1016/j.gastha.2022.11.006. PMC11308515 39132648

[50] Lindfors K, Koskinen O, Kaukinen K. An update on the diagnostics of celiac disease. Int Rev Immunol. 2011;30(4):185–196. 10.3109/08830185.2011.595854 21787224 21787224

[51] Reunamo A, Hannula M, Lindfors K, et al. Three-dimensional micro-computed tomographic imaging reveals early mucosal remodeling in individuals with potential celiac disease. Gastroenterology. 2026; 71(3):553–555. S0016-5085(26)06879-4. doi: 10.1053/j.gastro.2026.05.003. 42191048

[52] Popp A, Mäki M. Gluten-induced extra-intestinal manifestations in potential celiac disease—celiac trait. Nutrients. 2019;11(2):320. doi: 10.3390/nu11020320. PMC6412544 30717318

[53] Taavela J, Koskinen O, Huhtala H, et al. Validation of morphometric analyses of small-intestinal biopsy readouts in celiac disease. PLoS One. 2013;8(10):e76163. doi: 10.1371/journal.pone.0076163. PMC3795762 24146832

[54] Chelebian E, Avenel C, Wählby C. Combining spatial transcriptomics with tissue morphology. Nat Commun. 2025;16(1):4452. doi: 10.1038/s41467-025-58989-8. PMC12075478 40360467

[55] Wang JM, Hong R, Demicco EG, et al. Deep learning integrates histopathology and proteogenomics at a pan-cancer level. CR Med. 2023;4(9):101173. doi: 10.1016/j.xcrm.2023.101173. PMC10518635 37582371

[56] Pizurica M, Zheng Y, Carrillo-Perez F, et al. Digital profiling of gene expression from histology images with linearized attention. Nat Commun. 2024;15(1):9886. doi: 10.1038/s41467-024-54182-5. PMC11564640 39543087

[57] Ramón y Cajal S, Hümmer S, Peg V, et al. Integrating clinical, molecular, proteomic and histopathological data within the tissue context: tissunomics. Histopathology. 2019;75(1):4–19. doi: 10.1111/his.13828. PMC6851567 30667539

[58] Oittinen M, Popp A, Kurppa K, et al. Polycomb Repressive Complex 2 Enacts Wnt Signaling in Intestinal Homeostasis and Contributes to the Instigation of Stemness in Diseases Entailing Epithelial Hyperplasia or Neoplasia. Stem Cells. 2017;35(2):445–457. doi: 10.1002/stem.2479. 27570105

[59] Leblond CP, Walker BE. Renewal of cell populations. Physiol Rev. 1956;36(2):255–276. 10.1152/physrev.1956.36.2.255 13322651 13322651

[60] Barker N. Adult intestinal stem cells: critical drivers of epithelial homeostasis and regeneration. Nat Rev Mol Cell Biol. 2014;15(1):19–33. doi: 10.1038/nrm3721. 24326621

[61] Stamnaes J, Stray D, Du Pré MF, et al. IFN-γ is a direct driver of crypt hyperplasia in celiac disease. J Clin Invest. 2025;135(20):e194858. doi: 10.1172/JCI194858. PMC12520675 40828615

[62] Rondanelli M, Faliva MA, Gasparri C, et al. Micronutrients dietary supplementation advices for celiac patients on long-term gluten-free diet with good compliance: a review. Medicina (Kaunas). 2019;55(7):337. doi: 10.3390/medicina55070337. PMC6681258 31277328

[63] Syage JA, Kelly CP, Dickason MA, et al. Determination of gluten consumption in celiac disease patients on a gluten-free diet. Am J Clin Nutr. 2018;107(2):201–207. doi: 10.1093/ajcn/nqx049. 29529159

[64] Kreutz JM, Adriaanse MPM, van der Ploeg EMC, et al. Narrative review: nutrient deficiencies in adults and children with treated and untreated celiac disease. Nutrients. 2020;12(2):500. doi: 10.3390/nu12020500. PMC7071237 32075276

[65] Vici G, Belli L, Biondi M, et al. Gluten free diet and nutrient deficiencies: a review. Clin Nutr. 2016;35(6):1236–1241. doi: 10.1016/j.clnu.2016.05.002. 27211234

[66] Taavela J, Viiri K, Popp A, et al. Histological, immunohistochemical and mRNA gene expression responses in coeliac disease patients challenged with gluten using PAXgene fixed paraffin-embedded duodenal biopsies. BMC Gastroenterol. 2019;19(1):189. doi: 10.1186/s12876-019-1089-7. PMC6858741 31730447

[67] Taavela J, Viiri K, Välimäki A, et al. Apolipoprotein A4 defines the villus-crypt border in duodenal specimens for celiac disease morphometry. Front Immunol. 2021;12:713854. doi: 10.3389/fimmu.2021.713854. PMC8358775 34394117

[68] Dotsenko V, Le HH, Rajić S, et al. Therapeutic TG2 inhibition reverses systemic multiomic dysregulation in celiac disease. BMC Med. 2026;24(1):350. doi: 10.1186/s12916-026-04892-y. PMC13255354 42032651

[69] Vader W, Stepniak D, Kooy Y, et al. The HLA-DQ2 gene dose effect in celiac disease is directly related to the magnitude and breadth of gluten-specific T cell responses. Proc Natl Acad Sci U S A. 2003;100(21):12390–12395. doi: 10.1073/pnas.2135229100. PMC218768 14530392

[70] Koning F. Celiac disease: quantity matters. Semin Immunopathol. 2012;34(4):541–549. doi: 10.1007/s00281-012-0321-0. PMC3410019 22732901

[71] FastQC. A quality control tool for high throughput sequence data – ScienceOpen. Accessed July 21, ; 2026. https://www.scienceopen.com/book?vid=45910064-0c10-468b-8ac3-025edade0c9d.

[72] Chen S, Zhou Y, Chen Y, et al. fastp: an ultra-fast all-in-one FASTQ preprocessor. Bioinformatics. 2018;34(17):i884–i890. doi: 10.1093/bioinformatics/bty560. PMC6129281 30423086

[73] Martin M. Cutadapt removes adapter sequences from high-throughput sequencing reads. EMBnet j. 2011;17(1):10–12. doi: 10.14806/ej.17.1.200.

[74] Bolger AM, Lohse M, Usadel B. Trimmomatic: a flexible trimmer for Illumina sequence data. Bioinformatics. 2014;30(15):2114–2120. doi: 10.1093/bioinformatics/btu170. PMC4103590 24695404

[75] D A, Ca D, S F, et al. STAR: ultrafast universal RNA-seq aligner. Bioinformatics (Oxford, England). 2013;29(1). doi: 10.1093/bioinformatics/bts635. PMC3530905 23104886

[76] Kim D, Paggi JM, Park C, et al. Graph-based genome alignment and genotyping with HISAT2 and HISAT-genotype. Nat Biotechnol. 2019;37(8):907–915. doi: 10.1038/s41587-019-0201-4. PMC7605509 31375807

[77] HTSeq–a Python framework to work with high-throughput sequencing data - PubMed. Accessed July 21, ; 2026. https://pubmed.ncbi.nlm.nih.gov/25260700/. 10.1093/bioinformatics/btu638 PMC4287950 25260700

[78] Liao Y, Smyth GK, Shi W. featureCounts: an efficient general purpose program for assigning sequence reads to genomic features. Bioinformatics. 2014;30(7):923–930. doi: 10.1093/bioinformatics/btt656. 24227677

[79] Li H. Minimap2: pairwise alignment for nucleotide sequences. Bioinformatics. 2018;34(18):3094–3100. doi: 10.1093/bioinformatics/bty191. PMC6137996 29750242

[80] Ren J, Chaisson MJP. lra: A long read aligner for sequences and contigs. PLoS Comput Biol. 2021;17(6):e1009078. doi: 10.1371/journal.pcbi.1009078. PMC8248648 34153026

[81] Jain C, Rhie A, Hansen NF, et al. Long-read mapping to repetitive reference sequences using Winnowmap2. Nat Methods. 2022;19(6):705–710. doi: 10.1038/s41592-022-01457-8. PMC10510034 35365778

[82] Prjibelski AD, Mikheenko A, Joglekar A, et al. Accurate isoform discovery with IsoQuant using long reads. Nat Biotechnol. 2023;41(7):915–918. doi: 10.1038/s41587-022-01565-y. PMC10344776 36593406

[83] Tang AD, Soulette CM, van Baren MJ, et al. Full-length transcript characterization of SF3B1 mutation in chronic lymphocytic leukemia reveals downregulation of retained introns. Nat Commun. 2020;11(1):1438. doi: 10.1038/s41467-020-15171-6. PMC7080807 32188845

[84] SQANTI3: curation of long-read transcriptomes for accurate identification of known and novel isoforms - PubMed. Accessed. July 21, ; 2026. https://pubmed.ncbi.nlm.nih.gov/38509328/. 10.1038/s41592-024-02229-2 PMC11093726 38509328

[85] R D, N J, Tp S, et al. Normalization of RNA-seq data using factor analysis of control genes or samples. Nat Biotechnol. 2014;32(9):896–902. doi: 10.1038/nbt.2931. PMC4404308 25150836

[86] Love MI, Huber W, Anders S. Moderated estimation of fold change and dispersion for RNA-seq data with DESeq2. Genome Biol. 2014;15(12):550. doi: 10.1186/s13059-014-0550-8. PMC4302049 25516281

[87] Robinson MD, McCarthy DJ, Smyth GK. edgeR: a Bioconductor package for differential expression analysis of digital gene expression data. Bioinformatics. 2010;26(1):139–140. doi: 10.1093/bioinformatics/btp616. PMC2796818 19910308

[88] Wang X, Park J, Susztak K, et al. Bulk tissue cell type deconvolution with multi-subject single-cell expression reference. Nat Commun. 2019;10(1):380. doi: 10.1038/s41467-018-08023-x. PMC6342984 30670690

[89] Chen B, Khodadoust MS, Liu CL, et al. Profiling Tumor Infiltrating Immune Cells with CIBERSORT. Methods Mol Biol. 2018;1711:243–259. doi: 10.1007/978-1-4939-7493-1_12. PMC5895181 29344893

[90] Tang C, Sun Q, Zeng X, et al. Cell type-specific inference from bulk RNA-sequencing data by integrating single-cell reference profiles via EPIC-unmix. Genome Biol. 2025;26(1):395. doi: 10.1186/s13059-025-03847-5. PMC12636229 41267097

[91] Langfelder P, Horvath S. WGCNA: an R package for weighted correlation network analysis. BMC Bioinformatics. 2008;9:559. doi: 10.1186/1471-2105-9-559. PMC2631488 19114008

